# Automated Segmentation of Colorectal Tumor in 3D MRI Using 3D Multiscale Densely Connected Convolutional Neural Network

**DOI:** 10.1155/2019/1075434

**Published:** 2019-01-31

**Authors:** Mumtaz Hussain Soomro, Matteo Coppotelli, Silvia Conforto, Maurizio Schmid, Gaetano Giunta, Lorenzo Del Secco, Emanuele Neri, Damiano Caruso, Marco Rengo, Andrea Laghi

**Affiliations:** ^1^Department of Engineering, University of Roma Tre, Via Vito Volterra 62, 00146 Rome, Italy; ^2^Department of Radiological Sciences, University of Pisa, Via Savi 10, 56126 Pisa, Italy; ^3^Department of Radiological Sciences, Oncology and Pathology, University La Sapienza, AOU Sant'Andrea, Via di Grottarossa 1035, 00189 Rome, Italy

## Abstract

The main goal of this work is to automatically segment colorectal tumors in 3D T2-weighted (T2w) MRI with reasonable accuracy. For such a purpose, a novel deep learning-based algorithm suited for volumetric colorectal tumor segmentation is proposed. The proposed CNN architecture, based on densely connected neural network, contains multiscale dense interconnectivity between layers of fine and coarse scales, thus leveraging multiscale contextual information in the network to get better flow of information throughout the network. Additionally, the 3D level-set algorithm was incorporated as a postprocessing task to refine contours of the network predicted segmentation. The method was assessed on T2-weighted 3D MRI of 43 patients diagnosed with locally advanced colorectal tumor (cT3/T4). Cross validation was performed in 100 rounds by partitioning the dataset into 30 volumes for training and 13 for testing. Three performance metrics were computed to assess the similarity between predicted segmentation and the ground truth (i.e., manual segmentation by an expert radiologist/oncologist), including Dice similarity coefficient (DSC), recall rate (RR), and average surface distance (ASD). The above performance metrics were computed in terms of mean and standard deviation (mean ± standard deviation). The DSC, RR, and ASD were 0.8406 ± 0.0191, 0.8513 ± 0.0201, and 2.6407 ± 2.7975 before postprocessing, and these performance metrics became 0.8585 ± 0.0184, 0.8719 ± 0.0195, and 2.5401 ± 2.402 after postprocessing, respectively. We compared our proposed method to other existing volumetric medical image segmentation baseline methods (particularly 3D U-net and DenseVoxNet) in our segmentation tasks. The experimental results reveal that the proposed method has achieved better performance in colorectal tumor segmentation in volumetric MRI than the other baseline techniques.

## 1. Introduction

Colon and rectum are fundamental parts of the gastrointestinal (GI) or digestive system. The colon, which is also called the large intestine, starts from the small intestine and connects to the rectum. Its main function is to absorb minerals, nutrients, and water and remove waste from the body [1, 2]. According to recent cancer statistics, colorectal cancer is diagnosed as the second leading cause of cancer death in the United States [3].

**Figure 1:**
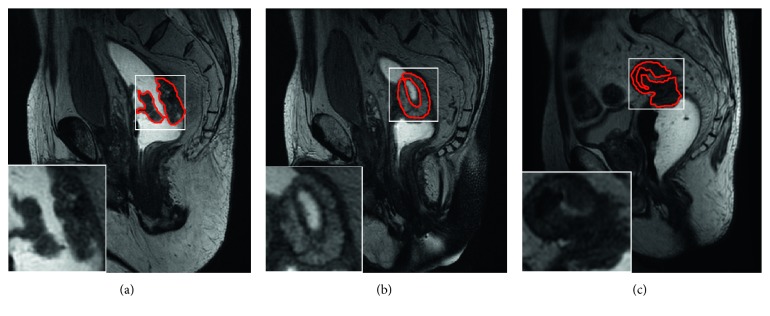
An illustration of colorectal tumor location, intensity, and size variation in a different slice of the same volume where the cancerous region is contoured by the red marker.

Nowadays, magnetic resonance imaging (MRI) is the most preferable medical imaging modality in primary colorectal cancer diagnosis for radiotherapy treatment planning [2, 4, 5]. Usually, the oncologist or radiologist delineates colorectal tumor regions from volumetric MRI data manually. This manual delineation or segmentation is time-consuming and laborious and presents inter- and intraobserver variability. Therefore, there exists a need for efficient automatic colorectal tumor segmentation methods in clinical radiotherapy practices to segment the colorectal tumor from large volumetric data, as this may save time and reduce human interventions. In contrast to natural images, medical imaging is generally more chaotic, as the shape of the cancerous regions may vary from slice to slice, as shown in Figure 1. Hence, automatic segmentation of the colorectal tumor is a very challenging task, not only because its size may be very small but also because of its rather inconsistent behavior in terms of shape and intensity distribution.

Lately, automatic segmentation of the colorectal tumor from volumetric MRI data based on atlas [6] and supervoxel clustering [7] has been presented with some good performance. Newly, deep learning-based approaches have been widely employed with impressive results in medical image segmentation [8–16]: Trebeschi et al. [8] have presented a deep learning-based automatic segmentation method to localize and segment the rectal tumor in multiparametric MRI by incorporating a fusion between T2-weighted (T2w) MRI and diffusion-weighted imaging (DWI) MRI. Despite their method displaying good performance, it is unclear whether only T2w modality, which provides more anatomy information than DWI modality, could be useful for colorectal tumor segmentation. Secondly, they applied their implementation on 2D data, as it is very common in real data, but medical data, such as CT (Computed Tomography) and MRI, are in 3D volumetric form. The 2D convolutional neural network (CNN) algorithms segment the volumetric MRI or CT data in a slice-by-slice sequence [9–11], where 2D kernels are used by aggregating axial, coronal, and sagittal planes in a one-to-one association, individually. Although these 2D CNN-based methods demonstrated great improvement in segmentation accuracy [17], the inherent 2D nature of the kernels limits their application when using volumetric spatial information. Based on this consideration, 3D CNN-based algorithms [12–16] have been recently presented, where 3D kernels are used instead of 2D to extract spatial information across all three volumetric dimensions. For example, Çiçek et al. [12] proposed a 3D U-net volume-to-volume segmentation network that is an extension of the 2D U-net [18]. 3D U-net used dual paths: an analysis path where features are abstracted, and a synthesis path or upsampling path where full resolution segmentation is produced. Additionally, 3D U-net established shortcut connections between early and later layers of the same resolution in both the analysis and synthesis paths. Chen et al. [13] presented a voxel-wise residual network (VoxResNet) that is an extension of 2D deep residual learning [19] to 3D deep network. VoxResNet provides a skip connection to pass features from one layer to the next layer. Even if these 3D U-net and VoxResNet provide several skip connections to make training easy, the presence of these skip connections creates a short path from the early layers to the last one and this may end up transforming the net into a very simple configuration, with the unwanted additional burden of producing a very high number of parameters to be adjusted during training. Huang et al. [20] introduced a DenseNet that extends the concept of skip connections in [18, 19] by constructing direct connections from every layer to the corresponding previous layers to ensure maximum gradient flow between layers. In [20], DenseNet was proven as an accurate and efficient method for the natural image classification. Yu et al. [16] proposed the densely connected volumetric convolutional neural network (DenseVoxNet) for volumetric cardiac segmentation which is an extended 3D version of DenseNet [20]. DenseVoxNet utilizes two dense blocks followed by pooling layers. The first block learns high-level feature maps, and the second block learns low-level feature maps; the latter is followed by a pooling layer that further reduces the resolution of the learned high-level feature maps in the first block. Finally, the high-resolution feature maps are restored by incorporating some deconvolution layers. In DenseVoxNet, early layers of the first block learn fine-scale features (i.e., high-level features) based on small receptive field, while coarse-scale features (i.e., low-level features) are learned by later layers of the second block with a larger receptive field. In short, fine-scale and coarse-scale features are learned in early and later layers, respectively, and this may reduce the network ability to learn multiscale contextual information throughout network, thus leading to suboptimal performance [21].

In this study, a novel method to overcome the abovementioned problems in 3D volumetric segmentation is presented. We propose a 3D multiscale densely connected convolutional neural network (3D MSDenseNet), a volumetric network that is an extension of the recently proposed 2D multiscale dense networks (MSDNet) for the natural image classification [22]. In summary, we have employed 3D MSDenseNet for the segmentation of the colorectal tumor, with the following contributions:

1. A multiscale training scheme with parallel 3D densely interconnected convolutional layers for two-dimensional depth and coarser scales is used where low- and high-level features are generated from each scale individually. A diagonal propagation layout is incorporated to couple the depth features with the coarser features from the first layer, thus maintaining local and global (multiscale) contextual information throughout the network to improve segmentation results efficiently.
2. The proposed network is based on volume-to-volume learning and interference, which eradicates computation redundancy.
3. The method is validated on colorectal tumor segmentation in 3D MR images, and it has attained outperformed segmentation results in comparison with previous baseline methods. From the encouraging results obtained with MR images, the proposed method could be applied for further applications of medical imaging.

## 2. Methods

**Figure 2:**
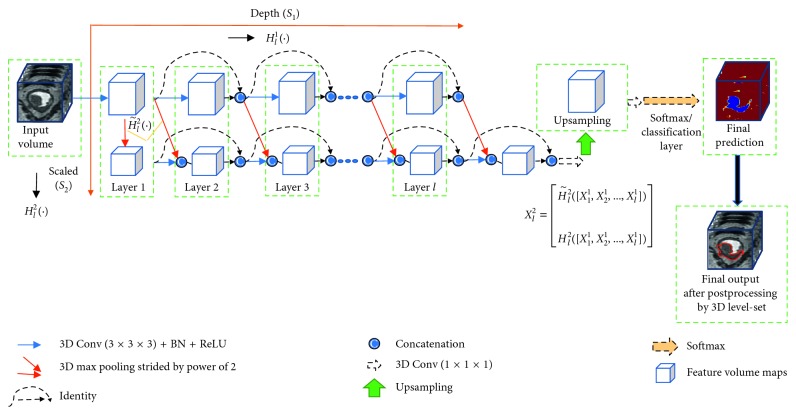
Block diagram of the proposed method.

Figure 2 represents an overview of our proposed methodology. We have extended the characterization of the multiscale densely connected network to colorectal tumor segmentation with 3D volume-to-volume learning fashion. The network is divided into two paths: depth path and scaled path. The depth path is similar to the dense network, which extracts the fine-scale features with high resolution. The scaled path is downsampled with a pooling layer of power 2. In this path, low-resolution features are learned. Furthermore, fine-scale features from depth are downsampled into coarse features via the diagonal path shown in Figure 2 and concatenated to the output of the convolution layer in the scaled path. By doing this, both local and global contextual information is incorporated in a dense network.

### 2.1. DenseNet: Densely Connected Convolutional Network

Generally, in feedforward CNN or ConvNet, the output of the *l*^th^ layer is represented as *X*_*l*_, which is obtained by mapping a nonlinear transformation *H*_*l*_ from the output of the preceding layer *X*_*l*−1_ such that

(1) $$\begin{array}{c} X_{l}=H_{l}\left(X_{l-1}\right), \end{array}$$

where *H*_*l*_ is composed of a convolution or pooling operation followed by a nonlinear activation function such as the rectified linear unit (ReLU) or batch normalization-ReLU (BN-ReLU). Recent works in computer vision have shown that a deeper network (i.e., with more layers) increases accuracy with better learning [20]. However, the performance of deeply modeled networks tends to decrease, and its training accuracy is saturated with the network depth increasing due to the vanishing/exploding gradient [20]. Later, Ronneberger et al. [18] solved this vanishing gradient problem in the deep network by incorporating skip connection, which propagates output features from layers of the same resolution in the contraction path to the output features from the layers in the expansion path. Nevertheless, this skip connection allows the gradient to flow directly from the low-resolution path to high-resolution path, which makes training easy, but this generally produces an enormous feature channel in every layer and lead network to adjust a large number of parameters during training. To overcome this problem, Huang et al. [20] introduced a densely connected network (DenseNet). The DenseNet extends the concept of skip connections by constructing a direct connection from every layer to its corresponding previous layers, to ensure maximum gradient flow between layers. In DenseNet, feature maps produced by the preceding layer were concatenated as an input to the advanced layer, thus providing a direct connection from any layer to the subsequent layer such that

(2) $$\begin{array}{c} X_{l}=H_{l}\left(\left[X_{l-1},X_{l-1},X_{l-1},\ldots ,X_{0}\right]\right), \end{array}$$

where [⋯] represents the concatenation operation. In [20], DenseNet has emerged as an accurate and efficient method for the natural image classification. Yu et al. [16] proposed densely connected volumetric convolutional neural network (DenseVoxNet) for volumetric cardiac segmentation which is an extended 3D version of DenseNet [20].

### 2.2. Proposed Method (3D MSDenseNet)

In 3D MSDenseNet, we have two interconnected levels, depth level and scaled level, for simultaneous computation of high- and low-level features, respectively. Let *X*_0_^1^ be an original input volume, and feature volume produced by layer *l* at scale *s* be represented as *X*_*l*_^*s*^. Considering two scales in the network (i.e., *s*_1_ and *s*_2_), we represent the depth level (horizontal path) and scaled level as *s*_1_ and *s*_2_ individually, as shown in Figure 2. The first layer is an inimitable layer where the feature map of the very first convolution layer is divided into respective scale *s*_2_ via pooling of stride of power 2. The high-resolution feature maps (*X*_*l*_^1^) in the horizontal path (*s*_1_) produced at subsequent layers (*l* > 1) are densely connected [20]. However, output feature maps of subsequent layers in the vertical path (i.e., coarser scale, *s*_2_) are results of concatenation of transformed features maps from previous layers in *s*_2_ and downsampled features maps from previous layers of *s*_1_, propagated as the diagonal way, as shown in Figure 2. In this way, output features of coarser scale *s*_2_ at layer *l* in our proposed network can be expressed as

(3) $$\begin{array}{c} X_{l}^{2}=\left[\begin{array}{c} {\tilde{H}}_{l}^{2}\left(\left[X_{1}^{1},X_{2}^{1},\ldots ,X_{l}^{1}\right]\right)\\ H_{l}^{2}\left(\left[X_{1}^{1},X_{2}^{1},\ldots ,X_{l}^{1}\right]\right) \end{array}\right], \end{array}$$

where [⋯] denotes the concatenation operator, ${\tilde{H}}_{l}^{2}\left(\cdot \right)$ represents those feature maps from finer scale *s*_1_ which are transformed by the pooling layer of stride of power 2 diagonally (as shown in Figure 2), and *H*_*l*_^2^(·) indicates those feature maps from coarser scale *s*_2_ transformed by regular convolution. Here, ${\tilde{H}}_{l}^{2}\left(\cdot \right)$ and *H*_*l*_^2^(·) have the same size of feature maps. In our network, the classifier only utilizes the feature maps from the coarser scale at layer *l* for the final prediction.

### 2.3. Contour Refinement with 3D Level-Set Algorithm

3D level-set based on the geodesic active contour method [23] is employed as a postprocessor to refine the final prediction of each network discussed above. 3D level-set adjusts the predicted tumor boundaries by incorporating prior information and a smoothing function. This 3D level-set method identifies a relationship between computation of geodesic distance curves and active contours. This relationship provides a precise detection of boundaries even in existence of huge gradient disparities and gaps. The level-set method based on the geodesic active contour is more elucidated with the mathematical derivations in [23, 24]. In order to simplify this algorithm, let *φ*(**P**_*l*_, *t*=0) be a level-set function which is initialized with the provided initial surface at *t* = 0. Here, **P**_*l*_ is the probability map yielded by each method. This probability map, **P**_*l*_, is employed as the starting surface to initialize the 3D level-set. Thereafter, the evolution of the level-set function regulates the boundaries of the predicted tumor. In the geodesic active contour, the partial differential equation is incorporated to evolve the level-set function [23] such that

(4) $$\begin{array}{c} \frac{\partial \varphi }{\partial t}=\alpha X\left(P_{l}\right)\cdot \nabla \varphi -\beta Y\left(P_{l}\right)\left|\nabla \varphi \right|+\gamma Z\left(P_{l}\right)\kappa \left|\nabla \varphi \right|, \end{array}$$

where **X**(·), **Y**(·), and **Z**(·) denote the convection function, expansion/contraction, and spatial modifier (i.e., smoothing) functions, respectively. In addition, *α*, *β*, and *γ* are the constant scalar quantities. The values of *α*, *β*, and *γ* bring the change in the above functions behavior. For example, negative values of *β* lead the initial surface to propagate in the outward direction with a given speed, while its positive value conveys the initial surface towards the inward direction. Evaluation of the level-set function is an iterative process; therefore, we have set the maximum number of iterations as 50 to stop the evolution process.

## 3. Experimental Setup

### 3.1. Experimental Datasets

The proposed method has been validated and compared on T2-weighted 3D colorectal MR images. Data were collected from two different hospitals: namely, Department of Radiological Sciences, Oncology and Pathology, University La Sapienza, AOU Sant'Andrea, Via di Grottarossa 1035, 00189 Rome, Italy; and Department of Radiological Sciences, University of Pisa, Via Savi 10, 56126 Pisa, Italy. MR data were acquired in a sagittal view on a 3.0 Tesla scanner without a contrast agent. The overall dataset consists of 43 volumes T2-weighted MRI, and each MRI volume consists of several slices, which are varied in number across subjects in the range 69∼122 and have dimension as 512 × 512 × (69∼122). The voxel spacing was varying from 0.46 × 0.46 × 0.5 to 0.6 × 0.6 × 1.2 mm/voxel across each subject. As the data have a slight slice gap, we did not incorporate any spatial resampling. The whole dataset was divided into training and testing sets for 100 repeated rounds of cross validation; i.e., 30 volumes were used for training and 13 for test until the combined results have given a numerically stable segmentation performance. The colorectal MR volumes were acquired in a sagittal view on a 3.0 Tesla scanner without a contrast agent. All MRI volumes went for preprocessing where they were normalized so that they have zero mean and unit variance. We cropped all the volumes with size of 195 × 114 × 61 mm. Furthermore, during training, the data were augmented with random rotations of 90°, 180°, and 270° in the sagittal plane to enlarge the training data. In addition, two medical experts using ITK-snap software [25, 26] manually segmented the colorectal tumor in all volumes. These manual delineations of tumors from each volume were then used as ground truth labels to train the network and validate it in the test phase.

### 3.2. Proposed Network Implementation

Our network architecture is composed of dual parallel paths, i.e., depth and scaled path, as illustrated in Figure 2, which achieves 3D end-to-end training by adopting the nature of the fully convolutional network. The depth path consists of eight transformation layers, and the scaled path consists of nine transformation layers. In each path, every transformation layer is composed of a BN, a ReLU followed by 3 × 3 × 3 convolution (Conv), by following the similar fashion of DenseVoxNet. Furthermore, a 3D upsampling block has been utilized like DenseVoxNet. Like DenseVoxNet, the proposed network uses the dropout layer with a dropout rate of 0.2 after each Conv layer to increase the robustness of the network against overfitting. Our proposed method has approximately 0.7 million as total parameters, which is much fewer than DenseVoxNet [16] with 1.8 million and 3D U-net [12] with 19.0 million parameters. We have implemented our proposed method in the Caffe library [27]. Our implementation code is available online at the Internet link http://host.uniroma3.it/laboratori/sp4te/teaching/sp4bme/documents/codemsdn.zip.

### 3.3. Networks Training Procedures

All the networks—3D FCNNs [15], 3D U-net [12], and DenseVoxNet [16]—were originally implemented in Caffe library [27]. For the sake of comparison, we have applied a training procedure which is very similar to that utilized by 3D U-net and DenseVoxNet.

Firstly, we randomly initialized the weights with a Gaussian distribution with *μ* = 0 and *σ* = 0.01. The stochastic gradient descent (SGD) algorithm [28] has been used to realize the network optimization. We set the metaparameters for the SGD algorithm to update the weights as batch size = 4, weight decay = 0.0005, and momentum = 0.05. We set the initial learning rate at 0.05 and divided by 10 every 50 epochs. Similar learning rate policy in DenseVoxNet, i.e., “poly,” was adopted for all the methods. The “poly”-learning rate policy changes the learning rate over each iteration by following a polynomial decay, where the learning rate is multiplied by the term (1 − (iteration/maximum_iterations))^power^ [29], where the term power was set as 0.9 and 40000 maximum iterations. Moreover, to ease GPU memory, the training volumes were cropped randomly with subvolumes of 32 × 32 × 32 voxels as inputs to the network and the major voting strategy [30] was incorporated to obtain final segmentation results from the predictions of the overlapped subvolumes. Finally, the softmax with cross-entropy loss was used to measure the loss between the predicted network output and the ground truth labels.

### 3.4. Performance Metrics

In this study, three evaluation metrics were used to validate and compare the proposed algorithm, namely, Dice similarity coefficient (DSC) [31], recall rate (RR), and average symmetric surface distance (ASD) [32]. These metrics are briefly explained as follows.

#### 3.4.1. Dice Similarity Coefficient (DSC)

The DSC is a widely explored performance metric in medical image segmentation. It is also known as overlap index. It computes a general overlap similarity rate between the given ground truth label and the predicted segmentation output by a segmentation method. DSC is expressed as

(5) $$\begin{array}{c} \text{DSC}\left(S_{\text{p}},S_{\text{g}}\right)=\frac{2\text{TP}}{\text{FP}+2\text{TP}+\text{FN}}=\frac{2\left|S_{\text{p}}\cap S_{\text{g}}\right|}{\left|S_{\text{p}}\right|+\left|S_{\text{g}}\right|}, \end{array}$$

where *S*_p_ and *S*_g_ are the predicted segmentation output and the ground truth label, respectively. FP, TP, and FN indicate false positives, true positives, and false negatives, individually. DSC gives a score between 0 and 1, where 1 gives the best prediction and indicates that the predicted segmentation output is identical to the ground truth.

#### 3.4.2. Recall Rate (RR)

RR is also referred as the true-positive rate (TPR) or sensitivity. We have utilized this term as the voxel-wise recall rate to assess the recall performance of different algorithms. This performance metrics measure misclassified and correctly classified tumor-related voxels. It is mathematically expressed as

(6) $$\begin{array}{c} \text{recall}=\frac{\text{TP}}{\text{TP}+\text{FN}}=\frac{\left|S_{\text{p}}\cap S_{\text{g}}\right|}{\left|S_{\text{g}}\right|}. \end{array}$$

It also gives a value between 0 and 1. Higher values indicate better predictions.

#### 3.4.3. Average Symmetric Surface Distance (ASD)

ASD measures an average distance between the sets of boundary voxels of the predicted segmentation and the ground truth and is mathematically given as

(7) $$\begin{array}{c} \text{ASD}\left(S_{\text{p}},S_{\text{g}}\right)=\frac{1}{\left|S_{\text{p}}\right|+\left|S_{\text{g}}\right|}\times \left(\underset{p_{k}\in S_{\text{p}}\;\;}{\sum }d\left(p_{k},S_{\text{g}}\right)+\underset{p_{g}\in S_{\text{g}}\;\;}{\sum }d\left(p_{g},S_{\text{p}}\right)\right), \end{array}$$

where *p*_*k*_ and *p*_*g*_ represent the *k*^th^ voxel from *S*_p_ and *S*_g_ sets, respectively. The function *d* denotes the point-to-set distance and is defined as *d*(*p*_*k*_, *S*_g_)=∑_*p*_*g*_∈*S*_g_ _‖*p*_*k*_ − *p*_*g*_‖, where ‖·‖ is the Euclidean distance. Lower values of ASD indicate higher closeness between the two sets, hence a better segmentation, and vice versa.

## 4. Experimental Results

In this section, we have experimentally evaluated the efficacy of multiscale end-to-end training scheme of our proposed method, where parallel 3D densely interconnected convolutional layers for two-dimensional depth and coarser scales paths are incorporated. Since this study is focused on the segmentation of tumors by 3D networks, the use of 2D networks is out of the scope of this paper. Nevertheless, we tried 2D networks in preliminary trials with a short set of image data. The 2D network was able to correctly recognize the tumor but could not segment the whole tumor accurately, especially in the presence of small size tumors.

In this work, the proposed network has been assessed on 3D colorectal MRI data. For more comprehensive analysis and comparison of segmentation results, each dataset was divided into ground truth masks (i.e., manual segmentation done by medical experts) and training and validation subsets. Quantitative and qualitative evaluations and comparisons with baseline networks are stated for the segmentation of the colorectal tumor. First, we have analyzed and compared the learning process of each method, like described in Section 4.1. Secondly, we have assessed the efficiency of each algorithm qualitatively; Section 4.2 presents a comparison of qualitative results. Finally, in Section 4.3, we have quantitatively evaluated the segmentation results yielded by each algorithm, using evaluation metrics as described below in Section 3.4.

### 4.1. Learning Curves

**Figure 3:**
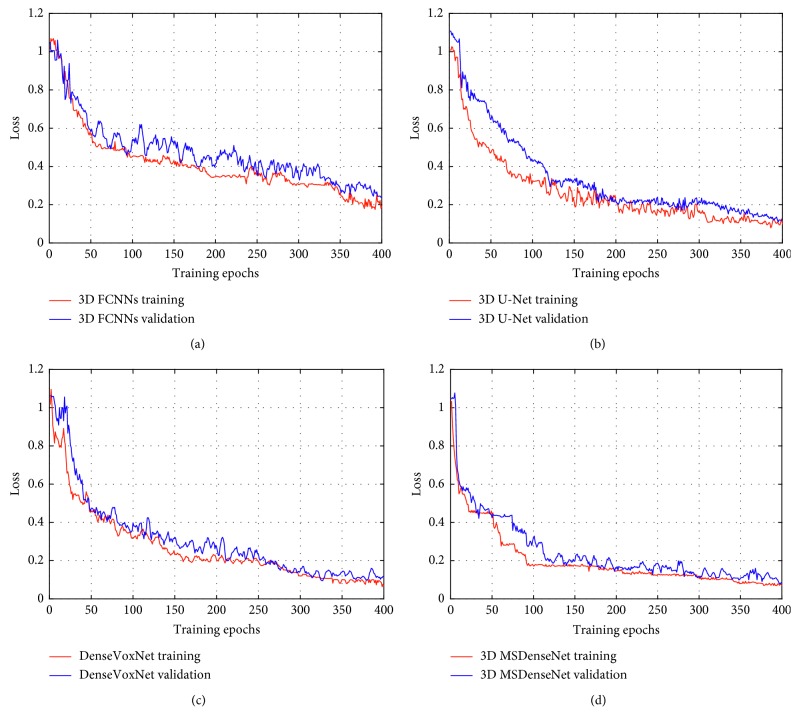
Comparison of learning curves of the examined methods. (a–d) Learning curves which correspond to 3D FCNNs, 3D U-net, DenseVoxNet, and the proposed 3D MSDenseNet methods, respectively.

The learning process of each method is illustrated in Figure 3, where loss versus training and loss versus validation are compared, individually, to some baseline methods. Figure 3 demonstrates that each method does not exhibit a serious overfitting as their validation loss consistently decreases along with decrement in training loss. Each method has adopted 3D fully convolutional architecture, where error back propagation is carried on pervoxel-wise strategy instead of the patch-based training scheme [33]. In other words, each single voxel is independently utilized as a training sample, which dramatically enlarges the training datasets and thus reduces the overfitting risk. In contrast to this, the traditional patch-based training scheme [33] needs a dense prediction (i.e., many patches are required) for each voxel in the 3D volumetric data, and thus the computation of these redundant patches for every voxel makes the network computationally too complex and impractical for volumetric segmentation.

After comparing the learning curves of 3D FCNNs (Figure 3(a)), 3D U-net (Figure 3(b)), and DenseVoxNet (Figure 3(c)), the 3D U-net and DenseVoxNet converge much faster with the minimum error rate than the 3D FCNNs. This demonstrates that both the 3D U-net and DenseVoxNet successfully overcome gradients vanishing/exploding problems through the reuse of the features of early layers till the later layers. On the contrary, it is also shown that there is no significant difference between learning curves of the 3D U-net and DenseVoxNet, although the DenseVoxNet attains a steady drop of validation loss in the beginning. It further proves that the reuse of the features from successive layers to every subsequent layer by DenseVoxNet, which propagates the maximum gradients instead of the skipped connections employed by 3D U-net, is able to propagate output features from layers with the same resolution in the contraction path to the output features from the layers in the expansion path. Furthermore, Figure 3(d) shows that the proposed method, that incorporates the multiscale dense training scheme, has the best loss rate among all the examined methods. It reveals that the multiscale training scheme in our method optimizes and speeds up the network training procedure. Thus, the proposed method has the fastest convergence with the lowest loss rate than all.

### 4.2. Qualitative Results

**Figure 4:**
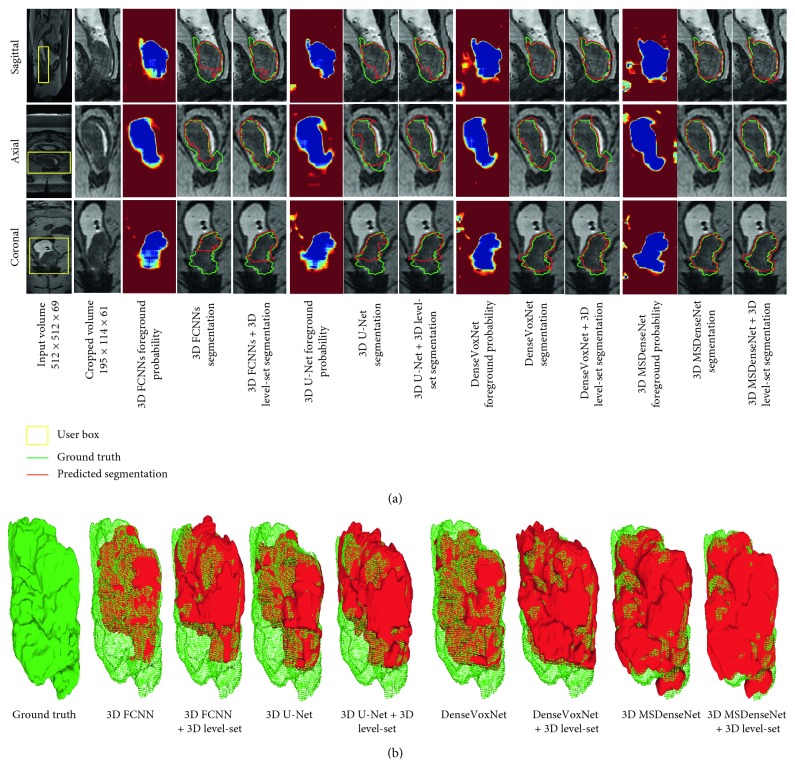
Qualitative comparison of colorectal tumor segmentation results produced by each method. In (a), from left to right columns are the raw MRI input volume and cropped volume, first three columns correspond to predicted probability by 3D FCNNs, and segmentation results by 3D FCNNs (red) and 3D FCNNs + 3D level set (red) overlapped with true ground truth (green), correspondingly. Similarly, second, third, and fourth three columns are related to predicted probability and segmentation results by rest of methods: 3D U-net (red), 3D U-net + 3D level set (red), DenseVoxNet (red), DenseVoxNet + 3D level set (red), 3D MSDensenet (red), and 3D MSDensenet + 3D level set (red), respectively. In (b), we have overlapped the 3D masks segmented by each method with the ground truth 3D mask. In (b), from left to right are ground truth 3D mask, overlapping of segmented 3D mask by 3D FCNNs (red), 3D FCNNs + 3D level set (red), 3D U-net (red), 3D U-net + 3D level set (red), DenseVoxNet (red), DenseVoxNet + 3D level set (red), 3D MSDensenet (red), and 3D MSDensenet + 3D level set (red) with the ground truth 3D mask (green points). The green points which are not covered by the segmentation results (red) of each method are referred as false negatives.

In this section, we report the qualitative results to assess the effectiveness of each segmentation method of the colorectal tumors.  Figure 4(a) gives a visual comparison of colorectal tumor segmentation results achieved from the examined methods. In Figure 4(a), from the left to right: the first two columns are the raw MRI input volume and its cropped volume, and the three following columns are related to the segmentation results produced by each method, where each column represents the predicted foreground probability, the initial colorectal segmentation results, and the refined segmentation results by the 3D level set. Moreover, the segmentation results produced by each method are outlined in red and overlapped with the true ground truth which is outlined in green. In Figure 4(b), we have overlapped the segmented 3D mask with the true ground truth 3D mask to visually evidence the false-negative rate in the segmentation results. It can be observed that the proposed method (3D MSDenseNet) outperforms the other methods, with the lowest false-negative rate, in respect to DenseVoxNet, 3D U-net, and 3D FCNNs. It is also noteworthy that the segmentation results obtained by each method significantly improves if a 3D level set is incorporated.

### 4.3. Quantitative Results

Table 1 presents the quantitative results of colorectal tumor segmentation produced by each method. The quantitative results are obtained by computing mean and standard deviation of each performance metric for all the 13 test volumes. We have initially compared the results obtained by each method without postprocessing by the 3D level set, considered here as baseline methods. Then, we present a comparison by incorporating the 3D level set as a postprocessor to refine the boundaries of the segmented results obtained by these baseline algorithms. In this way, we have got a total of eight settings, named as in the following: 3D FCNNs, 3D U-net, DenseVoxNet, 3D MSDenseNet, 3D FCNNs + 3D level set, 3D U-net + 3D level set, DenseVoxNet + 3D level set, and 3D MSDenseNet + 3D level set, respectively. Table 1 reveals that the 3D FCNNs have the lowest performance among all the metrics, followed by 3D U-net and DenseVoxNet, whereas the proposed method has maintained its performance by achieving the highest value of DSC and RR and the lowest value of ASD. When comparing the methods after postprocessing, every method has effectively improved their performance in the presence of the 3D level set: 3D FCNNs + 3D level set has improved DSC and RR as 16.44% and 15.23%, individually, and it reduced ASD to 3.0029 from 4.2613 mm. Similarly, 3D U-net + 3D level set and DenseVoxNet + 3D level set have attained improvements in DSC and RR as 5% and 5.97% and 4.99% and 4.29%, correspondingly. Also, they both have got a significant reduction in ASD as 3D U-net + 3D level set and DenseVoxNet + 3D level set reduce ASD to 2.8815 from 3.0173 and to 2.5249 from 2.7253, respectively. However, 3D MSDenseNet + 3D level set denotes a progress in DSC and RR as 2.13% and 2.42%, respectively, and it reduces ASD to 2.5401 from 2.6407. Nevertheless, the 3D MSDenseNet + 3D level-set method could not attain a significant improvement by utilizing the postprocessing step but still outperforms among all. Considering both qualitative and quantitative results, it can be observed that the addition of the 3D level set as a postprocessor improves the segmentation results of each method.

## 5. Discussion

In this work, we have tested the method 3D FCNNs + 3D level set [15], devised from mostly the same authors as this paper, together with two further prominent and widely explored volumetric segmentation algorithms, namely, 3D U-net [12] and 3D DenseVoxNet [16], for volumetric segmentation of the colorectal tumor from T2-weighted abdominal MRI. Furthermore, we have extended their ability for the colorectal tumor segmentation task by the incorporating 3D level set in their original implementations. In order to improve the performance, we have proposed a novel algorithm based on 3D multiscale densely connected neural network (3D MSDenseNet). Many studies were carried out in the literature to develop techniques for medical image segmentation; they are mostly based on geometrical methods to address the hurdles and challenges for the segmentation of medical imaging, including statistical shape models, graph cuts, level set, and so on [34]. Recently, level set-based segmentation algorithms were commonly explored approaches for medical image segmentations. Generally, they utilize energy minimization approaches by incorporating different regularization terms (smoothing terms) and prior information (i.e., initial contour etc.) depending on the segmentation tasks. Level set-based segmentation algorithms take advantage of their ability to vary topological properties of segmentation function [35], so it becomes attractive. However, they always require an initial appropriate contour initialization to segment a desired object. This initial contour initialization requires an expert user intervention in the medical image segmentation. In addition, since medical images have disordered intensity distribution and show high variability (among imaging modalities, slices, etc.), a segmentation based on statistical models of intensity distribution is not successful. More precisely, level set-based approaches, given their simple appearance model [36], and lack of generalization ability and transferability are in some cases unable to learn alone the chaotic intensity distribution in medical images. Currently, CNNs deep learning-based approaches (i.e., CNNs) have been successfully explored in the medical imaging domain, specifically for classification, detection, and segmentation tasks. Usually, deep learning-based approaches learn a model by extracting features deeply from intricate structures and patterns from well-defined big training datasets where the trained model are used for prediction. In contrast to level set-based approaches, deep learning-based approaches can learn appearance models automatically from the big training data, which improves its transferability and generalization ability. However, deep learning-based approaches are not capable to provide an explicit way to incorporate a function to have the tendency of delivering regularization or smoothing terms like the level-set function has. Therefore, in order to take the advantages of both level-set and deep learning into account, we have incorporated 3D level set in each method that we used in our task.

Moreover, traditional CNNs are 2D in nature and were designed especially for 2D natural images, whereas medical images like MRI or CT are inherently in the 3D form. Generally, these 2D CNNs with 2D kernels have been used for medical image segmentation where volumetric segmentation was performed in a slice-by-slice sequential order. Such 2D kernels are not able to completely make use of volumetric spatial information by sharing spatial information among the three planes. A 3D CNN architecture that utilizes 3D kernel which simultaneously share spatial information among three planes can offer a more effective solution.

Another challenge of 3D CNN involves controlling the hurdles in network optimization when the network goes deeper. Deeper networks are more prone to get risk of overfitting, due to vanishing of gradients in advance layers. This has been confirmed in this work. From the segmentation results produced by 3D FCNNs, we can see from Figure 4 that how the patterns/gradients have been lessened. In order to preserve the gradients in next layers when the network goes deeper, 3D U-net and DenseVoxNet reuse the features from early to next layers. In this way, 3D U-net overcomes the vanishing gradient problem in deep network by incorporating skip connection, which propagates output features from layers of the same resolution in the contraction path to the output features from the layers in the expansion path. Nevertheless, such a skip connection allows the gradient to flow directly from the low-resolution path to the high-resolution one, which makes the training easy, but this generally produces a very high number of feature channels in every layer and leads to adjust a big number of parameters during training. To overcome this problem, the DenseVoxNet extends the concept of skip connections by constructing a direct connection from every layer to its corresponding previous layers, to ensure the maximum gradient flow between layers. In simple words, feature maps produced by the preceding layer are concatenated as an input to the advanced layer, thus providing a direct connection from any layer to the subsequent layer. Our results have proven that the direct connection strategy of DenseVoxNet provides better segmentation than the skip connection strategy of 3D U-net. However, DenseVoxNet has a deficit as the network individually learns high-level and low-level features in early and later layers; this limits the network to learn multiscale contextual information throughout the network and may lead the network to a poor performance. The network we have proposed provides a multiscale dense training scheme where high-resolution and low-resolution features are learned simultaneously, thus maintaining maximum gradients throughout the network. Our experimental analysis reveals that reusing features through multiscale dense connectivity produces an effective colorectal tumor segmentation. Nevertheless, although the proposed method has obtained better performance in colorectal tumor segmentation, the algorithm presented herein has higher variance in DSC and RR values, compared with the other methods, as shown in Table 1. It evidences that the proposed algorithm may not be able to compare contrast variations in a cancerous region and variations of slice gap along the *z*-axis among the datasets. A better normalization and superresolution method with more training samples might then be required to circumvent this problem.

## 6. Conclusion

In this research work, a novel 3D fully convolutional network architecture (3D MSDenseNet) is presented for accurate colorectal tumor segmentation in T2-weighted MRI volumes. The proposed network provides a dense interconnectivity among the horizontal (depth) and vertical (scaled) layers. In this way, finer (i.e., high-resolution features) and coarser (low-resolution features) features are coupled in a two-dimensional array of horizontal and vertical layers, and thus, features of all resolutions are produced from the first layer on and maintained throughout the network. However, in other network (viz. traditional CNN, 3D U-net, or DenseVoxNet) coarse level features are generated with an increasing network depth. The experimental results show that the multiscale scheme of our approach has attained the best performance among all. Moreover, we have incorporated the 3D level set algorithm within each method, as a postprocessor that refines the segmented prediction. It has been also shown that adding a 3D level set increases the performance of all deep learning-based approaches. In addition, the proposed method, due to its simple network architecture, has a total number of parameters consisting of approximately 0.7 million, which is much fewer than DenseVoxNet with 1.8 million and 3D U-net with 19.0 million parameters. As a possible future direction, the proposed method could be further validate on other medical volumetric segmentation tasks.

## Figures and Tables

**Table 1:** Quantitative comparison of colorectal tumor segmentation results.

Methods	Performance metrics		
	DSC	RR	ASD (mm)
3D FCNNs [15]	0.6519 ± 0.0181	0.6858 ± 0.1017	4.2613 ± 3.1603
3D U-net [12]	0.7227 ± 0.0128	0.7463 ± 0.0302	3.0173 ± 3.0133
DenseVoxNet [16]	0.7826 ± 0.0146	0.8061 ± 0.0187	2.7253 ± 2.9024
3D MSDenseNet (proposed method)	**0.8406** **±** **0.0191**	**0.8513** **±** **0.0201**	2.6407 ± 2.7975
3D FCNNs + 3D level set [15]	0.7591 ± 0.0169	0.7903 ± 0.0183	3.0029 ± 2.9819
3D U-net + 3D level set	0.8217 ± 0.0173	0.8394 ± 0.0193	2.8815 ± 2.6901
DenseVoxNet + 3D level set	0.8261 ± 0.0139	0.8407 ± 0.0177	**2.5249** **±** **2.8004**
3D MSDenseNet + 3D level set (proposed method)	**0.8585** **±** **0.0184**	**0.8719** **±** **0.0195**	2.5401 ± 2.402

## Data Availability

The T2-weighted MRI data used to support the findings of this study are restricted by the ethical board of Department of Radiological Sciences, University of Pisa, Via Savi 10, 56126 Pisa, Italy, and Department of Radiological Sciences, Oncology and Pathology, University La Sapienza, AOU Sant'Andrea, Via di Grottarossa 1035, 00189 Rome, Italy, in order to protect patient privacy. Data are available from Prof. Andrea Laghi (Department of Radiological Sciences, Oncology and Pathology, University La Sapienza, AOU Sant'Andrea, Via di Grottarossa 1035, 00189 Rome, Italy) and Prof. Emanuele Neri (Department of Radiological Sciences, University of Pisa, Via Savi 10, 56126 Pisa, Italy) for researchers who meet the criteria for access to confidential data.
